# Density influences the heritability and genetic correlations of fish behaviour under trawling‐associated selection

**DOI:** 10.1111/eva.13279

**Published:** 2021-08-04

**Authors:** Amélie Crespel, Toby Miller, Anita Rácz, Kevin Parsons, Jan Lindström, Shaun Killen

**Affiliations:** ^1^ Institute of Biodiversity, Animal Health and Comparative Medicine University of Glasgow Glasgow UK; ^2^ Department of Biology University of Turku Turku Finland; ^3^ Department of Genetics Eötvös Loránd University Budapest Hungary

**Keywords:** activity, fisheries, genetic correlation, heritability, indirect selection, sociability

## Abstract

Fishing‐associated selection is one of the most important human‐induced evolutionary pressures for natural populations. However, it is unclear whether fishing leads to heritable phenotypic changes in the targeted populations, as the heritability and genetic correlations of traits potentially under selection have received little attention. In addition, phenotypic changes could arise from fishing‐associated environmental effects, such as reductions in population density. Using fish reared at baseline and reduced group density and repeatedly harvested by simulated trawling, we show that trawling can induce direct selection on fish social behaviour. As sociability has significant heritability and is also genetically correlated with activity and exploration, trawling has the potential to induce both direct selection and indirect selection on a variety of fish behaviours, potentially leading to evolution over time. However, while trawling selection was consistent between density conditions, the heritability and genetic correlations of behaviours changed according to the population density. Fishing‐associated environmental effects can thus modify the evolutionary potential of fish behaviour, revealing the need to use a more integrative approach to address the evolutionary consequences of fishing.

## 1 INTRODUCTION

Harvest‐associated selection is one of the most important human‐induced evolutionary pressures for natural populations (Darimont et al., 2015; Hendry et al., 2017). Phenotypic changes in life‐history traits (such as reduced body size‐at‐age and/or earlier maturation) have been observed in many targeted populations, threatening their resilience (Coltman, 2008; Jorgensen et al., 2007; Law, 2000; Marty et al., 2015; Sharpe & Hendry, 2009). While individual behaviour is the first line of defence to human‐induced stressors, evidence for harvest‐associated behavioural changes has so far been limited. In a fishing context, however, recent experimental studies have highlighted the potential for the evolution of behaviour (Diaz Pauli & Sih, 2017; Heino et al., 2015), with a timidity syndrome (i.e. when an exploited population becomes shyer than unexploited populations) for populations exploited by passive gears (Arlinghaus et al., 2017). As changes in individual behaviour could have strong ecological consequences for populations, communities and ecosystems (Arlinghaus et al., 2017; Diaz Pauli & Sih, 2017), it is essential to understand how fisheries‐induced evolution could impact a larger variety of behaviour. In particular, the mechanisms by which the phenotypic changes may occur are not yet understood, as evolution through modification of the underlying genetic variation in populations remains uncertain.

For evolution to occur, harvesting must select on specific phenotypes with heritable variation (Law, 2000). The targeted populations should thus express phenotypic variability with respect to capture vulnerability. Depending on the fishing gear, fisheries can induce selection on various fish traits in addition to their life history (Diaz Pauli & Sih, 2017; Hollins et al., 2018). Passive gears (e.g. angling or traps), which depend on fish to approach and interact with the deployed gear, have generally been reported to select against bold, more explorative and active individuals, while active gear (e.g. trawls or seines) relying on fish capacity to escape may be more selective for physiological performance (such as swimming capacity or anaerobic metabolism) (Arlinghaus et al., 2017; Biro & Post, 2008; Diaz Pauli & Sih, 2017; Killen et al., 2015). It is less known whether active gears such as trawls have the potential to exert selection on fish behaviour. The few studies on the topic have revealed that active gears could select for fish timidity and reduced activity (Diaz Pauli et al., 2015; Hollins et al., 2020). Other behaviours, especially those involving sociality, could also be under selection (Diaz Pauli & Sih, 2017), especially considering that many active gears exploit the schooling and social characteristics of targeted species (Guerra et al., 2020). To understand the possible evolutionary responses of targeted populations, it is crucial to determine the evolutionary potential (heritability and genetic correlations) of the traits under fishing selection. While behaviours are known to display flexibility, they can still possess significant heritability and genetic correlations with other traits (Ferrari et al., 2016), potentially leading to indirect selection. Significant heritability for boldness or aggressiveness has been reported in fish (Ariyomo et al., 2013; Dingemanse et al., 2009; Ferrari et al., 2016) but so far, the heritability and genetic correlations for behaviours potentially under fishing selection are still largely unknown. A better understanding of this is needed to estimate the evolutionary consequences of fishing and better inform sustainable strategies for fisheries management.

Harvest‐associated plasticity could also occur as fishing induces confounding environmental effects that influence phenotypic development (Ratner & Lande, 2001). For example, a major environmental change caused by intense harvesting would be the removal of a substantial proportion of individuals from the populations, inducing density‐dependent effects. A reduction in the population density could therefore have important effects on individual behaviour by changing the developmental trajectories, social interactions and competitive environment within the targeted population (Amundsen et al., 2006; Kavanagh & Olney, 2006). Such environmental effects could modify overall behavioural expression in the remaining population but also modify which individuals have a selective advantage in a new population structure, potentially shifting the selectivity of fishing gear. Different genotypes may thus be selected against depending on the prevailing population density. In addition, as individual behaviours can be plastic, different sets of underlying genes may be expressed in the new density environment, completely shifting the heritability and genetic correlations of behaviours (Campbell et al., 2017; Pigliucci, 2005). Such gene‐by‐environment (GxE) interactions may then alter the evolutionary potential of fish behaviour, ultimately constraining or facilitating the evolutionary response of fishing in the remaining population (Nussey et al., 2007; Wilson et al., 2006). Population density could thus represent an important determinant of harvest‐induced evolution that has been theorized (Eikeset et al., 2016; Kuparinen et al., 2014) but not addressed in empirical studies so far.

The aim of the present study was to investigate the role of direct harvest selection, population density reduction, as well as their interaction, on the evolutionary potential of fish behaviour in experimental populations under trawling pressure. Notably, the behavioural traits measured in this study should not be interpreted in terms of animal personality, which implies consistency across contexts and over time (Castanheira et al., 2013). We were interested to determine whether trawling can induce selection on specific dimensions of fish behaviour and whether these behaviours were heritable. We also examined the potential for population density reduction to shift fish behavioural expression or selection and evolutionary potential due to fishing via gene‐by‐environment interactions (G×E). A better understanding of the effects of both direct gear selection and associated population density reductions on fish behaviour is critical to fully address the potential ecological and evolutionary consequences of fishing.

## 2 MATERIAL AND METHODS

### 2.1 Experimental populations

The use of experimental animals for the study of fisheries impacts has been previously advocated (Conover & Baumann, 2009) as it allows running small‐scaled fishing simulations and investigating animal responses or environmental contexts that are not possible in the wild. By behaving similarly as the majority of the larger targeted species by fisheries (in terms of exploration, sociability and shoaling), zebrafish represent ideal surrogate species (Thambithurai et al., 2018). A semi‐wild population (about five generations in captivity) of zebrafish (*Danio rerio*) sourced from rearing ponds in Malaysia (JMC Aquatics, Sheffield) was used as a study population. The genetic diversity of the population used was in the range of the genetic diversity in natural zebrafish populations (expected heterozygosity, He = 0.25) (Whiteley et al., 2011). After transfer to the University of Glasgow and quarantined for few weeks, 24 adult fish were bred in a controlled factorial (North Carolina II) design where four groups of three males were reciprocally crossed with three females (i.e. nine families per group) producing 36 full‐sib families nested into a half‐sib structure in June 2017. Such breeding design allows for the estimation of narrow‐sense heritability, minimizing any nongenetic bias in our estimates. When the larvae fully hatched and became free swimmers (4 days postfertilization), each family was split equally into two developmental density conditions: baseline density (according to standard protocols, 60 larvae/L; Avdesh et al., 2012) and reduced density (half of baseline density, 30 larvae/L). Each family was then maintained separately in three 3‐L tanks supplied with recirculating 28°C dechlorinated freshwater in a 13‐L:11‐D photoperiod (one used for the baseline density and two for the reduced density per family, i.e. 36 tanks in baseline density and 72 tanks in reduced density in total). The larvae were fed four times a day using a combination of commercial food (TetraMin baby, ZM fry food, Zebrafeed, Novo Tom) and live *Artemia* nauplii, taking care of giving half ration for the tanks with half fish density. At six months, 10 fish from each family and developmental density condition (i.e. 360 fish per density) were randomly taken and individually tagged using visible implant elastomers (VIE, North West Marine Technology, WA, USA). Each family was then distributed equally (one or two fish per family per tank) into 13.5‐L tanks supplied with recirculating 28°C dechlorinated freshwater. All the families were thus mixed in each tank. The baseline density fish were housed in eight 13.5‐L tanks and the reduced density fish in sixteen 13.5‐L tanks (i.e. the fish were housed in 24 tanks in total). The density conditions were then adjusted to 6 fish/L (baseline density according to standard protocols, Avdesh et al., 2012) and 3 fish/L (reduced density) using nonexperimental fish from the same families when needed. The fish were fed twice a day using commercial food (TetraMin Tropical Flakes, ZM small granula) and live *Artemia* nauplii, again taking care of giving half of the ration to the reduced density fish. Less than 1% mortality was observed during the rearing period. Before every manipulation, the fish were fasted for 24 h.

### 2.2 Behaviour assessment

**FIGURE 1:**
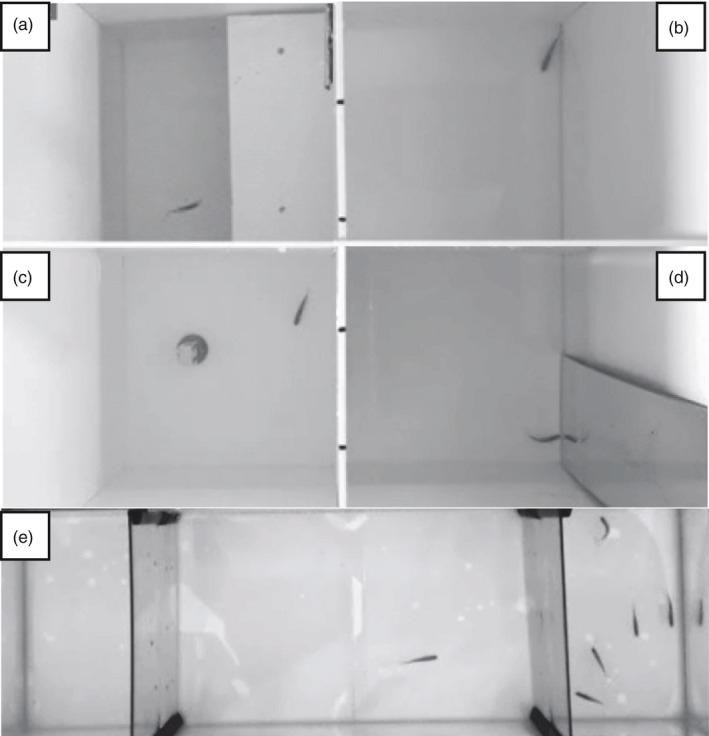
Photographs of the different behavioural assays. (a) Shelter assay; (b) open field assay; (c) novel object assay; (d) mirror assay; (e) sociability assay

Behavioural assays (shelter, open field, novel object and mirror assays; Figure 1) were conducted in 16 individual square plastic tanks (17 × 17 cm) filled to a 5 cm depth with fully oxygenated freshwater thermoregulated at 28°C. The shelter assay was conducted using an opaque rectangle (17 × 8 cm) attached at the surface of the water on one side of the tanks. The open field assay was conducted in the open field tanks by removing the shelter. The novel object assay was conducted by introducing a yellow Lego^®^ brick at the centre of the tanks. The mirror assay was conducted by introducing a mirror (8.5 × 30 cm) on one side of the tanks. Four webcams (Logitech D Pro C920) were attached at 1 m above the level of water to record fish behaviour using the iSpy software (iSpyConnect). The entire setup was shielded from surrounding disturbances behind a plastic blind.

Each day, 16 fish were assessed for the set of behaviours. The fish were placed in the tanks containing the shelter and left to acclimate for 10 min. Fish behaviour in the shelter assay was then recorded for 20 min. The shelter was then removed, and the fish behaviour in the open field assay was recorded for 20 min. The novel object was then introduced into tanks, and the fish behaviour in the novel object assay was recorded for 20 min. The novel object was then removed, and fish were left to acclimate in the empty tanks for 20 min. The mirror was then introduced into the tanks, and the fish behaviour in the mirror assay was recorded for 10 min. The mass and fork length of the fish were then measured.

After a minimum of one week of recovery from the first set of behavioural assays, the fish were assessed for sociability. Sociability assays were conducted in four individual rectangular glass tanks divided in three sections, comprising a central focus section (32 × 19 cm) and two side sections (13 × 19 cm) separated by transparent acrylic, filled to a 10 cm depth with fully oxygenated freshwater at 28°C (Figure 1). Between each trial, half of the water was changed to maintain the desired temperature and oxygen levels. Two webcams (Logitech D Pro C920) were attached at 1 m above the level of the water to record fish behaviour using the iSpy software (iSpyConnect). The entire setup was shielded from surrounding disturbances behind curtains.

At the beginning of the trials, a group of six fish (three males and three females unfamiliar with the focal fish) were placed randomly on one side section of each tank, and the other sides remained empty. After 5 min of acclimation for these groups of fish, the focal fish was introduced in the middle of the central focus section of the tanks within a transparent plastic cylinder. After 5 min of acclimation for the focal fish, the transparent cylinder was removed and fish behaviour was recorded for 20 min. The mass and fork length for each fish was then measured.

Videos were analysed using Ethovision XT11 (Noldus 2001). In the shelter assay, total distance moved (cm), time spent moving (s), mean speed while in motion (cm/s), number of time out of the shelter, total time spent out of the shelter (s) and time of first exit of the shelter (s) were determined. In the open field assay, total distance moved (cm), time spent moving (s), mean speed while in motion (cm/s) and average distance to the centre of the arena (cm) were determined. In the novel object assay, total distance moved (cm), time spent moving (s), mean speed while in motion (cm/s), average distance to the centre of the arena (cm) and average distance to novel object (cm) were determined. In the mirror assay, number of time biting the mirror, time spent biting the mirror (s), time of first mirror bite (s), time spent in proximity (within 7 cm) of the mirror (s) and time of first entrance in proximity of the mirror (s) were determined. In the sociability assay, the average distance of the focal fish to conspecifics (cm) was determined. The measure of the behavioural traits was not intended to quantify the personality of each individual fish, but to investigate behaviour responses at the family and population levels. The average length of the fish was 3.01 ± 0.01 cm in the baseline density and 3.15 ± 0.01 cm in the reduced density.

### 2.3 Trawling simulations

After all fish were assessed for behaviours, the entire population from both developmental density conditions (*N* = 360 per density) were repeatedly exposed to trawling simulations, over six fishing trials, to mimic commercial fisheries that gradually harvest fish over time. The scaled‐down trawling simulations were conducted in a 90‐L swimming tunnel (Loligo Systems) filled with fully oxygenated freshwater thermoregulated to 28°C. A 30‐cm small‐scaled model trawl net with codend and escape routes on the upper sides of the net mouth (designed by the Fisheries and Marine Institute of Memorial University of Newfoundland) was fixed inside the swimming tunnel for the simulations. The entire setup was shielded from surrounding disturbances behind black curtains.

Each fishing trial was comprised of several simulated fishing events. At the beginning of each simulation event, fish were acclimated by groups of 16 for 20 min at a water velocity of 4 cm/s in front of the net mouth hidden by an opaque divider. After the acclimation, the divider was removed and the water velocity was increased to 50 cm/s (upper limit of sustainable swimming similarly as in actual trawling, Winger et al., 2010) over 30 s. The simulation event lasted 10 min during which the time a fish got captured by falling inside the net was recorded. At the end of the 10 min, the location of the fish (captured inside the codend, captured inside the net, escaped in front of the net mouth, escaped behind the net) was also recorded. The location and time of capture were then used to determine the fish vulnerability to the trawling simulation. Once the entire population from each developmental density condition passed through the first fishing trial (22 trawling simulation events), the 20% most vulnerable fish in each density condition (*N* = 72 per density) were identified, based on the shortest time until capture, and removed from the following fishing trials. A new series of simulation events was then conducted on the remaining population in each density. Every week for six weeks, a fishing trial was conducted (six fishing trials consisting of 150 fishing events in total across densities and tanks), each time identifying the 20% of fish that were most vulnerable to capture in each density condition. At the end of the six trials, the 20% least vulnerable fish were identified in each density (*N* = 72 per density), that is the fish that were able to escape every trawling simulation. A vulnerability score from 1 to 7 was then attributed to the fish depending on the number of fishing trials the fish were able to escape. The fish with the score 1 were thus the most vulnerable fish, being caught at the first fishing trial, while the fish with the score 7 were the least vulnerable fish, never captured over the six fishing trials.

### 2.4 Statistics

Principal component analysis (PCA) using scaled data in ‘prcomp’ (using a correlation matrix) was conducted on the behavioural data sets to extract more integrative behaviour indices. Two behaviour subdata sets were used in two PCA analyses to explore intra‐individual (PCA‐Intra) and interindividual (PCA‐Inter) behaviour (Table 1). The PCA‐Intra included the variables from the three behaviour assays (shelter, open field and novel object assays) reflecting how the fish explore their surrounding environment. The PCA‐Inter was conducted to explore interindividual behaviour by including the variables from the two behaviour assays (mirror and sociability assays) capturing how the fish interact with other fish. The number of principal component axes retained for the analysis was based on eigenvalues >1 but also on a‐posteriori evaluation of the quantity and the type of variance explained. The first two principal component axes from each PCA were thus retained for further analysis as they were representative of most of the variance observed (PC1‐Intra and PC2‐Intra from the intra‐individual PCA explained 34.8% and 14.4%, respectively, eigenvalues 5.22 and 2.15, respectively, and PC1‐Inter and PC2‐Inter from the interindividual PCA explained 40.5% and 20.6%, respectively, eigenvalues 2.43 and 1.23, respectively). Despite PC3‐Intra having an eigenvalue of 1.18, it was not included in the analysis as it was integrating redundant information to PC2‐Intra from the biological perspective (mainly including average distance to the centre of the arena in the open field and novel assays, as well as average distance to the novel object). In addition, number of mirror bites and distance to conspecifics were used as single variables (as proxies for aggression and asociability, respectively), to more precisely examine trawling selection on these social interaction traits without the effects of the other variables included in the PCA analysis as such social characteristics were expected to be particularly targeted by active gear.

**TABLE 1:** Individual traits measured during the different behavioural assays and their inclusion and impact in the principal component analysis (PCA)

Behavioural assay	Behaviour measured	PCA analysis	Impact on PC1	Impact on PC2
Shelter assay	Total distance moved	PCA‐Intra	0.37	−0.25
	Time spent moving	PCA‐Intra	0.36	−0.30
	Mean speed while in motion	PCA‐Intra	−0.13	0.10
	Number of time out of the shelter	PCA‐Intra	0.34	−0.21
	Total time spent out of the shelter	PCA‐Intra	0.34	−0.31
	Time to first exit of the shelter	PCA‐Intra	−0.20	0.12
Open field assay	Total distance moved	PCA‐Intra	0.31	0.28
	Time spent moving	PCA‐Intra	0.32	0.12
	Mean speed while in motion	PCA‐Intra	0.06	0.32
	Average distance to the centre of the arena	PCA‐Intra	−0.10	−0.29
Novel assay	Total distance moved	PCA‐Intra	0.29	0.28
	Time spent moving	PCA‐Intra	0.32	0.18
	Speed	PCA‐Intra	0.01	0.13
	Average distance to the centre of the arena	PCA‐Intra	−0.15	−0.39
	Average distance to the novel object	PCA‐Intra	−0.13	−0.34
Mirror assay	Number of mirror bites	PCA‐Inter	0.48	−0.41
	Time spent biting the mirror	PCA‐Inter	0.43	−0.52
	Time of first mirror bite	PCA‐Inter	−0.51	−0.18
	Time spent in proximity of the mirror	PCA‐Inter	0.35	0.43
	Time of first entrance in proximity of the mirror	PCA‐Inter	−0.45	−0.39
Sociability assay	Average distance to conspecifics	PCA‐Inter	0.00	0.44

General linear mixed models were used to analyse the different fish behaviours (PC1‐Intra, PC2‐Intra, PC1‐Inter, PC2‐Inter, number of bites and distance to conspecifics) initially with sex, density and capture vulnerability (continuous), as well as their interactions, the quadratic of capture vulnerability (as visual inspection of the data indicated the potential for a curvilinear relationship) and length as covariate, all fitted as fixed effects and days of measurements fitted as random effect. For each variable, the models were then reduced and selected by comparing the models’ AICs. PC1‐Intra and PC2‐Intra final models included the main effects of sex, density and vulnerability as fixed effects and days as random effects. The PC1‐Inter and number of bites final models included the main effects of density, vulnerability and the quadratic of vulnerability. The PC2‐Inter and distance to conspecifics final models included the main effects of sex, density and vulnerability. Data normality and homogeneity of variances were verified according to the analysis of the distribution of the models residuals and Levene tests, respectively. A posteriori Tukey's tests were used for mean comparison. All the statistical analyses were performed using R v4.0.0 (R Core Team, 2020).

### 2.5 Quantitative genetics

To determine the evolutionary potential (heritability and genetic correlations) of the measured behaviours, our breeding design was used to fit an animal model (Lynch & Walsh, 1998) using the software ASReml (Gilmour et al., 2015). The variance components of the different behaviours (PC1‐Intra, PC2‐Intra, PC1‐Inter, PC2‐Inter, number of bites and distance to conspecifics) were estimated separately within each developmental density condition by restricted maximum likelihood (REML) using a mixed model that included sex as fixed effect, the individual random additive genetic effect linked to the pedigree structure and the residuals. The common environment effect of family members was investigated by including the family as an additional random effect in the model, but was then removed from the models as it was not statistically significant for any of the traits and did not impact the statistical significance and estimates of heritability. The model for PC1‐Intra and PC2‐Intra also included the days of measurements as fixed effects. The total phenotypic variance (*V*_P_) of each trait was decomposed into additive variance (*V*_A_) and residual variance (*V*_R_) allowing the estimation of the narrow‐sense heritability (*h* ^2^) as the ratio of the additive variance to the total phenotypic variance (*h* ^2^ = *V*_A_/*V*_P_). The significance of each additive genetic variances within densities was tested by doing a pairwise comparison between the full model and a null model where all variance was residual (no random additive genetic effects included in the model), using a likelihood ratio test. Genetic (*r*_G_) and phenotypic (*r*_P_) correlations among behaviours within each developmental density condition were estimated using bivariate models including two behavioural traits as response variables. Genetic and phenotypic correlations between density conditions were also estimated for each behaviour measured using bivariate models in which the fish behaviours from the two density conditions were included as response variables. The genetic correlations were not estimated for behaviours without significant additive genetic variance. A likelihood ratio test was used to evaluate the significance of the genetic components (additive genetic (co)variance) estimated within and between densities by comparing the full model with a null model (including the random effects but in which the (co)variance was set to zero). The similarity between the genetic variance estimates for the same trait between density treatments was tested by comparing the full model with a constrained model (including the random effects but in which the estimates were set to be equal) using a likelihood ratio test.

## 3 RESULTS

### 3.1 Principal component analysis (PCA) of fish behaviour

Based on a minimum factor loading of 0.3 when interpreting the dimensions, the first dimension of the PCA‐Intra (Dim1—PC1‐Intra—34.8% variance explained) was used as an activity index, the higher the index the more active the fish, while the second dimension (Dim2—PC2‐Intra—14.4% variance explained) was used as an exploration index, the higher the index the more curious the fish. The first dimension of the PCA‐Inter (Dim1—PC1‐Inter—40.5% variance explained) was used as an aggression index, the higher the index the more aggressive the fish, while the second dimension (Dim2—PC2‐Inter—20.6% variance explained) was used as an asociability index, the higher the index the less social the fish.

### 3.2 Trawling selection on fish behaviour

**FIGURE 2:**
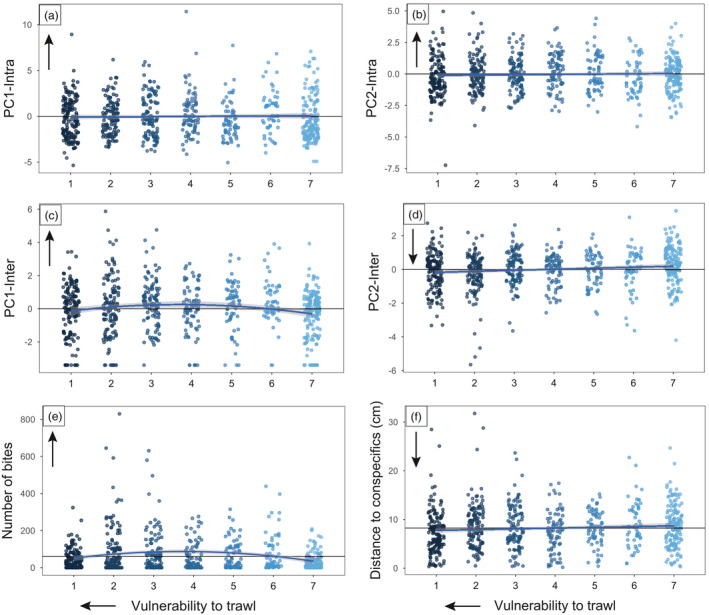
Behavioural changes across weeks of simulated trawling selection. (a) PC1‐Intra (activity index), (b) PC2‐Intra (exploration index), (c) PC1‐Inter (aggression index), (d) PC2‐Inter (asociability index), (e) number of mirror bites and (f) Distance to conspecifics (cm). The arrows represent the direction of the traits. Higher values represent higher activity (PC1‐Inta, a), higher exploration (PC2‐Intra, b), higher aggression (PC1‐Inter, c), lower sociability (PC2‐Inter, d), higher aggression (number of bites, e) and lower sociability (distance to conspecifics, f). Higher values in vulnerability to trawl represent lower vulnerability: vulnerability to trawl from 1 to 6 represents the fish captured at each trawling trail, the fish from the vulnerability score 1 being more vulnerable than fish from the vulnerability score 6, vulnerability score 7 represents the fish that escaped all the simulations, so the fish the least vulnerable. The zero black line represents the average behaviour for the population, while the blue line represents the behavioural response model. The shaded areas around the blue lines correspond to 95% confidence intervals

Fish intra‐individual behaviour, in terms of both activity (PC1‐Intra) and exploration (PC2‐Intra) indices, was not selected by the trawling simulations in either developmental density condition (PC1‐Intra: GLMM, *F*_1,674_ = 0.08, *p *= 0.77; PC2‐Intra: GLMM, *F*_1,682_ = 0.07, *p *= 0.80; Figure 2). No interactions between vulnerability and density were retained in the models, and no main effect of density was observed (PC1‐Intra: GLMM, *F*_1,66_ = 0.57, *p *= 0.45; PC2‐Intra: GLMM, *F*_1,56_ = 0.25, *p *= 0.62). Only a sex effect was observed for PC2‐Intra (GLMM, *F*_1,676_ = 7.36, *p *= 0.007), with males being more exploratory than females.

In contrast, fish interindividual behaviour was under selection by the trawling simulations regardless of population developmental density, as no interactions between vulnerability and density were retained. During the first four weeks of capture, PC1‐Inter (aggression index) was higher when vulnerability decreased, while after the fourth week, PC1‐Inter of escaped fish was lower than those that were captured with fish having escaped all trawling simulations (vulnerability score 7) being the least aggressive (GLM, capture vulnerability *F*_1,681_ = 8.22, *p *= 0.004, quadratic of capture vulnerability *F*_1,681_ = 10.16, *p *= 0.001; Figure 2). This curvilinear response of aggression was supported by the analysis of the number of mirror bites, with more bites given by the fish with lower vulnerability during the first four weeks of capture and then a lower number of bites for the fish with lower vulnerability afterwards (GLM, capture vulnerability *F*_1,715_ = 24.10, *p *< 0.001, quadratic of capture vulnerability *F*_1,715_ = 29.02, *p *< 0.001). PC2‐Inter (asociability index) was also higher in fish that were less vulnerable to capture, revealing lower sociability for those fish (GLM, *F*_1,681_ = 5.85, *p *= 0.016; Figure 2). Overall, a sex effect was observed for PC2‐Inter with the males being less social than the females (GLM, *F*_1,681_ = 13.20, *p *= 0.0003). A similar effect of fishing vulnerability was observed for distance to conspecifics, with less vulnerable fish being more distant from conspecifics and so less social (GLM, *F*_1,681_ = 4.28, *p *= 0.04). No main effect of density was observed on either PC1‐Inter, PC2‐Inter, number of bites or distance to conspecifics (PC1‐Inter: GLM, *F*_1,681_ = 0.29, *p *= 0.59; PC2‐Inter: GLM, *F*_1,681_ = 1.11, *p *= 0.29; number of bites: GLM, *F*_1,715_ = 2.30, *p *= 0.13; distance to conspecifics: GLM, *F*_1,681_ = 0.71, *p *= 0.40).

### 3.3 Heritability and genetic correlations of fish behaviour under baseline density

Under baseline density, behaviours not under selection from the trawling simulations (i.e. PC1‐Intra and PC2‐Intra) displayed significant genetic variance and heritability ($X_{1}^{2}$ = 15.62, *p *< 0.001; $X_{1}^{2}$ = 4.82, *p *= 0.03, respectively). However, behaviours that were under selection by the trawling simulations (PC1‐Inter, PC2‐Inter and number of bites) did not ($X_{1}^{2}$ = 0.228, *p *= 0.63; $X_{1}^{2}$ = 0.13, *p *= 0.03; $X_{1}^{2}$ = 0.72, *p *= 0.99, respectively) (Table 2), except the distance to conspecifics which displayed a significant genetic variance and heritability ($X_{1}^{2}$ = 20.35, *p *< 0.001). Fish length also displayed significant genetic variance and heritability ($X_{1}^{2}$ = 15.07, *p *< 0.001).

**TABLE 2:** Heritability and genetic correlations of the different behaviours within and between density conditions

	Baseline density			Reduced density			Between density				Similarity *p* values
	*h* ^2^	VA	*p* values	*h* ^2^	VA	*p* values	Gen corr	*p* values	Phen corr	*p* values	
Length (cm)	**0.20 (0.09)**	**0.008 (0.004)**	**<0.001**	**0.13 (0.07)**	**0.004 (0.003)**	**0.005**	**0.58 (0.35)**	**0.015**	0.05 (0.05)	0.58	**0.017**
PC1‐Intra	**0.21 (0.10)**	**0.91 (0.45)**	**<0.001**	**0.27 (0.11)**	**1.37 (0.60)**	**<0.001**	**0.99 (0.13)**	**<0.001**	**0.19 (0.06)**	**<0.001**	**<0.001**
PC2‐Intra	**0.11 (0.07)**	**0.21 (0.14)**	**0.03**	0.07 (0.06)	0.15 (0.13)	0.14	–	–	0.05 (0.06)	0.28	**0.043**
PC1‐Inter	0.02 (0.04)	0.06 (0.12)	0.63	0.02 (0.03)	0.04 (0.08)	0.53	–	–	**0.12 (0.05)**	**0.04**	0.14
PC2‐Inter	0.01 (0.03)	0.0002 (0.0387)	0.72	**0.17 (0.08)**	**0.20 (0.11)**	**<0.001**	–	–	0.04 (0.05)	0.18	**<0.001**
Number of bites	0.01 (0.01)	0.0003 (0.0001)	0.99	0.03 (0.04)	0.02 (0.02)	0.38	–	–	0.02 (0.05)	0.65	0.13
Distance to conspecifics (cm)	**0.27 (0.12)**	**5.49 (2.67)**	**<0.001**	**0.20 (0.09)**	**3.93 (1.92)**	**<0.001**	**0.99 (0.23)**	**<0.001**	**0.09 (0.06)**	**0.03**	**<0.001**

Note

Estimates of heritability [*h* ^2^ (SE)] and genetic variance [VA (SE)] in the zebrafish reared under baseline or reduced density, as well as genetic [Gen corr (SE)] and phenotypic [Phen corr (SE)] correlation between density for fork length, PC1‐Intra (activity index), PC2‐Intra (exploration index), PC1‐Inter (aggression index), PC2‐Inter (asociability index), number of mirror bites and distance to conspecifics. *p* values for baseline and reduced density were obtained by likelihood ratio tests between a full model and a model with the variance set to be null (i.e. only residual variance). *p* values for the correlations between densities were obtained from a likelihood ratio tests between a full model and a model where the random (co)variance was constrained to zero. The genetic correlations were not estimated for behaviours without significant additive variance in at least one of the density conditions and were represented by a dash. *p* values for similarity were obtained by likelihood ratio tests between a full model and a model where the (co)variance was constrained to be equal and indicate the difference in the genetic variance between density conditions. Significant parameters are in bold. Mean ± SEM.

Significant phenotypic correlations were observed among the PC1‐Intra and all the other behaviours measured (except the number of mirror bites), meaning a higher activity level being phenotypically correlated with higher exploration (PC2‐Intra) and aggression (PC1‐Inter) and lower sociability (measured as higher PC2‐Inter and distance to conspecifics) (Table 3). PC1‐Inter showed a positive phenotypic correlation with mirror bites but was also negatively phenotypically correlated to the asociability estimates (PC2‐Inter and distance to conspecifics) as well as fish length. PC2‐Inter and distance to conspecifics were also positively phenotypically correlated, and both were correlated with fish length. Despite these phenotypic correlations, the behaviours were not always genetically correlated (Table 3). PC1‐Intra (activity index) was only strongly positively genetically correlated with distance to conspecifics (*r*_G_ = 0.88, *p *= 0.006). Also, while not phenotypically correlated, PC2‐Intra (exploration index) and distance to conspecifics were strongly genetically positively correlated (*r*_G_ = 0.89, *p *= 0.01). Length was not significantly genetically correlated with any behaviour.

**TABLE 3:** Pairwise genetic and phenotypic correlation matrix of the different behaviours within density conditions

	Length	PC1‐Intra	PC2‐Intra	PC1‐Inter	PC2‐Inter	Number of bites	Distance to conspecifics
BD							
Length		−0.02 (0.06) *p* = 0.42	0.01 (0.06) *p* = 0.75	−0.11 (0.06) *p* = 0.03	0.10 (0.05) *p* = 0.03	−0.07 (0.05) *p* = 0.15	0.13 (0.06) *p* = 0.005
PC1‐Intra	0.09 (0.35) *p* = 0.80		0.13 (0.06) *p* = 0.01	0.19 (0.05) *p* < 0.001	0.23 (0.06) *p* < 0.001	−0.06 (0.01) *p* = 0.06	0.10 (0.06) *p* = 0.04
PC2‐Intra	−0.37 (0.36) *p* = 0.33	0.66 (0.33) *p* = 0.10		0.06 (0.05) *p* = 0.15	0.06 (0.05) *p* = 0.13	0.02 (0.05) *p* = 0.66	0.04 (0.06) *p* = 0.36
PC1‐Inter	–	–	–		−0.09 (0.06) *p* = 0.02	0.76 (0.01) *p* < 0.001	−0.10 (0.05) *p* = 0.04
PC2‐Inter	–	–	–	–		−0.50 (0.04) *p* < 0.001	0.51 (0.04) *p* < 0.001
Number of bites	–	–	–	–	–		−0.06 (0.01) *p* = 0.06
Distance to conspecifics	−0.06 (0.35) *p* = 0.88	0.88 (0.18) *p* = 0.006	0.89 (0.22) *p* = 0.01	–	–	–	
RD							
Length		0.07 (0.06) *p* = 0.86	0.02 (0.05) *p* = 0.22	0.01 (0.05) *p* = 0.69	0.07 (0.05) *p* = 0.32	−0.04 (0.05) *p* = 0.53	0.01 (0.05) *p* = 0.84
PC1‐Intra	−0.16 (0.36) *p* = 0.66		0.16 (0.05) *p* = 0.01	0.11 (0.05) *p* = 0.03	0.09 (0.06) *p* = 0.04	0.05 (0.05) *p* = 0.32	0.09 (0.06) *p* = 0.04
PC2‐Intra	–	–		−0.02 (0.05) *p* = 0.68	0.21 (0.05) *p* < 0.001	−0.09 (0.05) *p* = 0.03	0.04 (0.06) *p* = 0.27
PC1‐Inter	–	–	–		0.10 (0.05) *p* = 0.02	0.73 (0.02) *p* < 0.001	0.10 (0.05) *p* = 0.02
PC2‐Inter	0.59 (0.36) *p* = 0.16	−0.53 (0.27) *p* = 0.09	–	–		−0.39 (0.06) *p* = 0.01	0.45 (0.04) *p* < 0.001
Number of bites	–	–	–	–	–		0.01 (0.05) *p* = 0.59
Distance to conspecifics	0.50 (0.38) *p* = 0.23	0.71 (0.22) *p* = 0.02	–	–	0.76 (0.19) *p* = 0.01	–	

Note

Estimates of phenotypic (above diagonal) and genetic (below diagonal) correlations with standard error (SE) in the zebrafish reared into baseline density (BD) or reduced density (RD) for fork length, PC1‐Intra (activity index), PC2‐Intra (exploration index), PC1‐Inter (aggression index), PC2‐Inter (asociability index), number of mirror bites and distance to conspecifics. Genetic correlations were not estimated for behaviours without significant additive variance and were represented by a dash. Red cells indicate significant positive correlation while blue cells indicate significant negative correlations. Mean ± SEM.

### 3.4 Density effect on the heritability and genetic correlations of fish behaviour

Reduced density changed the heritability and genetic correlations underlying behaviour. Under reduced density, PC2‐Intra did not display significant genetic variance and heritability ($X_{1}^{2}$ = 2.21, *p *= 0.14), while PC2‐Inter genetic variance and heritability were significant ($X_{1}^{2}$ = 12.27, *p *< 0.001) (Table 2). PC1‐Intra and distance to conspecifics still displayed significant genetic variance and heritability under reduced density ($X_{1}^{2}$ = 31.95, *p *< 0.001; $X_{1}^{2}$ = 17.67, *p *< 0.001, respectively) while PC1‐Inter and number of bites still did not ($X_{1}^{2}$ = 0.39, *p *= 0.53; $X_{1}^{2}$ = 0.78, *p *= 0.38, respectively). Length also showed significant genetic variance and heritability under reduced density ($X_{1}^{2}$ = 7.61, *p *= 0.005).

Only PC1‐Intra and distance to conspecifics had a strong significant genetic correlation between densities (*r*_G_ = 0.99, *p *< 0.001). When comparing the genetic variance of traits between the densities, length, PC2‐Intra and distance to conspecifics had significantly lower genetic variance under reduced density while PC1‐Intra and PC2‐Inter had significantly higher genetic variance under reduced density. PC1‐Inter and number of mirror bites were not different between densities.

Different phenotypic correlations were observed among behaviours under a reduced density. PC2‐Intra was positively phenotypically correlated to PC2‐Inter and negatively phenotypically correlated to the number of bites (Table 3), meaning more exploratory fish were less social and less aggressive. In addition, PC1‐Inter (aggression index) was positively phenotypically correlated with asociability estimates (PC2‐Inter and distance to conspecifics). Fish length was no longer phenotypically correlated to any behaviour. The genetic correlation among the different behaviours also differed from the baseline density condition (Table 3). While PC1‐Intra (activity index) was still highly genetically positively correlated to distance to conspecifics (*r*_G_ = 0.71, *p *= 0.02), distance to conspecifics was not genetically correlated to PC2‐Intra (exploration index) as PC2‐Intra was not possessing significant additive variance. However, distance to conspecifics was strongly positively genetically correlated to PC2‐Inter (asociability index, *r*_G_ = 0.76, *p *= 0.01). Fish length was still not genetically correlated to any behaviour.

## 4 DISCUSSION

While the potential for an evolutionary response to harvesting remains a central question, the present study suggests that trawling can induce selection on heritable fish behaviours, but the strength of the evolutionary response will depend on the behaviour of interest and also the fish population developmental density. Experimental trawling imposed direct selection on social behaviour in both densities, especially on scores for PC2‐Inter and distance to conspecifics, with individuals that were more likely to escape being less social. As distance to conspecifics was heritable under both baseline and reduced density, and PC2‐Inter was heritable under reduced density, the results suggest that trawling has the potential to induce the evolution of sociability over time. In addition, because distance to conspecifics shares genetic covariance with fish PC1‐Intra and PC2‐Intra (activity and exploration indices), indirect selection on these behaviours seems possible even if they are not directly selected upon by fishing. However, the genetic variance and correlations of the behaviours were different depending on developmental density. Distance to conspecifics did not share genetic variance with fish PC2‐Intra under reduced density, minimizing the potential for indirect selection between these two traits under these conditions. Overall, this study reveals that trawling can lead to both direct selection and indirect selection on various heritable behavioural traits and that population developmental density can influence the evolutionary potential of this selection.

Behaviours associated with fish social interactions (aggressiveness and sociability), but not fish intra‐individual behaviour (activity and exploration indexes), were under direct selection by the trawling simulations under both density conditions. Active fishing gear can thus directly target specific individual behaviours. Trawling selection on fish social behaviour has been speculated because of the ways in which trawls target and exploit fish schooling behaviour (Diaz Pauli & Sih, 2017; Godo et al., 1999; Heino & Godo, 2002; Hollins et al., 2018; Winger et al., 2004). Our findings align with observations of actual trawls, where fish often follow conspecifics into the net or maintain position with swimming schoolmates until they fatigue and fall into the net (Underwood et al., 2015; Winger et al., 2010). However, the degree of selectivity on aggression seems to depend on the fishing pressure, represented in the current study by the number of trawling trials. We can only speculate as to the potential mechanisms underlying this effect, but it is possible that the most aggressive fish were initially more likely to escape repeated capture attempts if they can outcompete the less aggressive fish for access to escape routes. However, as fishing events continue and less aggressive fish are selectively removed from the population, there may be a shift in the selective advantage of this behaviour resulting in fish less aggressive being more likely to escape. The absence of direct selection on PC1‐Intra and PC2‐Intra (activity and exploration) behaviour contradicts previous studies. Activity and exploration have previously been reported to be directly targeted during active trawling simulations, leading to fish that are more timid and less active having a selective disadvantage (Andersen et al., 2018; Diaz Pauli et al., 2015; Hollins et al., 2020). This selection was explained as shyer fish being more likely to be captured as they would freeze instead of fleeing to escape when facing a trawl. As no direct selection was observed in the present study, it seems that this may not be the case for every species or that direct selection on these traits may be sensitive to specific fishing procedures or environmental conditions. Overall, our study highlights that trawls may be more likely to directly select fish according to their social behaviours than their activity and exploration behaviour. It should be noted, however, that our study only looked at the stage of fishing where fish are already facing the trawl mouth. Activity and exploration behaviour could be under direct selection at earlier stages of the fishing process involving habitat selection, space use and gear encounter (Hollins et al., 2018; Olsen et al., 2012). It is also possible that some learning across the fishing simulations might have occurred during our experiment. After the first fishing trial, fish with a higher capacity for learning (either to avoid the trawl or to find escape routes) may have been less likely to be captured. Further investigation is needed to estimate the effects of learning in fishing selection, but learning is believed to be relevant in actual trawl fisheries, where fish can escape trawls (or be released after capture) and potential encounter trawls multiple times throughout their lives (Suuronen, 2005). Finally, our trawling simulations used the same group size for each density. While this allows investigating the long‐term density developmental effect on fish behaviour and vulnerability to capture, it would be interesting to examine the ability of fish to evade capture in trawls when they are in various group sizes, as previously observed for fish exposed to traps (Thambithurai et al., 2018).

By possessing significant genetic variance and heritability, especially when quantified in terms of distance to conspecifics in the sociability assay, sociability has the potential to evolve in populations exposed to trawling pressure. In the present study, we estimated the additive genetic variance based on a semi‐factorial North Carolina breeding design which allows more precise narrow‐sense heritability estimation. Therefore, nongenetic effects are minimized in our estimates. The evolution of sociability could have strong repercussions for the resilience of the targeted population as the fish stock could become less social, potentially leading to altered collective behaviours and reduced social cohesion (Arlinghaus et al., 2017; Diaz Pauli & Sih, 2017; Guerra et al., 2020). In addition, such evolution could reduce the genetic diversity present across generations, ultimately leading the population towards an evolutionary trap and reducing its capacity to adapt to further harvesting pressure or additional environmental perturbations. The heritability of sociability measured here was in the same range as heritability of sociability measured previously in wild sticklebacks (range 0.20–0.42; Dingemanse et al., 2009). Distance to conspecifics also shared a genetic variance with PC1‐Intra and PC2‐Inta under baseline density, leading to indirect trawling selection for activity and exploration. Heritability for PC1‐Intra and PC2‐Intra (indexes of activity and exploration, heritability 0.21–0.27 and 0.07–0.11, respectively) was in a similar range or lower than previously observed for boldness in other populations or fish species (wild sticklebacks: 0.14–0.33, Dingemanse et al., 2009; domesticated zebrafish: 0.49–0.90, Ariyomo et al., 2013; semi‐wild European Seabass: 0.45, Ferrari et al., 2016). However, it should be noted that the behavioural assays to measure boldness can vary across studies, potentially affecting the heritability estimates. A trawling‐associated reduction in sociability across generations could therefore lead to fish being also more active and exploratory, even if no direct selection was observed for these traits. This indirect selection could partially explain the previous observations that active gear may target more timid individuals as reported by Diaz Pauli and Sih (2017). Evolution of the population towards higher activity and exploration could not only affect the resilience of the population but also produce a trophic cascade and perturb community and ecosystem functioning (Andersen et al., 2018; Arlinghaus et al., 2017; Diaz Pauli et al., 2019; Diaz Pauli & Sih, 2017). This is especially true if fish become both less social and more active, thus losing the antipredator benefits of grouping while also increasing their encounter rates with predators. Moreover, the reductions in activity level within populations due to evolution are expected to strongly erode vulnerability to capture, ultimately reducing the effectiveness of fisheries (Andersen et al., 2018; Arlinghaus et al., 2017). The combination of the direct selection on sociability and indirect selection on activity and exploration might then hinder the potential of recovery for the exploited populations (Salinas et al., 2012). It is, however, important to carefully distinguish between direct selection and indirect selection, as they would have to be managed differently with regard to fisheries regulations. With direct selection, for example, changes to fishing gears or practices may alleviate selection on particular traits that are associated with individual likelihood of being captured. Understanding and limiting correlated selection is more complex, requiring knowledge of the genetic correlations among traits and the path through which selection is happening.

The heritability and genetic correlations of behaviour changed depending on the population developmental density, although trawling selection on behaviour was similar between densities. Differences in the heritability and genetic correlations among traits between densities could be explained by the differing genetic variances and covariances within each density. Indeed, PC2‐Inter had a significantly higher genetic variance while fish length and PC2‐Intra had a significantly lower genetic variance under reduced density. PC2‐Inter was thus heritable under reduced density, which would strengthen the evolutionary responses towards reduced sociability fish in response to fishing under these conditions. However, PC2‐Intra and distance to conspecifics did not possess genetic covariance under the reduced density, impeding the indirect selection towards more exploratory fish. Such differences in genetic variance and covariance between densities might be the result of density‐dependent G×E interactions. Depending on the developmental density of the population, fish may experience differing levels of social interaction or competition (Amundsen et al., 2006; Laursen et al., 2013), potentially generating behavioural plasticity and shifts in underlying genes involved in the expression of these behaviours. It has previously been reported that developmental density may change the expression of gene related to immune function and stress response (Yarahmadi et al., 2016). As fishing will be accompanied by a reduction in population density within and across generations, such G×E interactions are likely to occur in actual fisheries. On the one hand, such G×E effects could be advantageous if they relax indirect selection on traits such as exploration as the density of the population reduces. On the other hand, due to the loss of genetic variance for fish exploration, G×E effects could reduce the pace of population recovery after the release of fishing pressure, as previously modelled (Kuparinen et al., 2014; Marty et al., 2015), density‐dependent effects, including compensatory responses, being reported to have major importance for population evolution and recovery under fishing pressure (Eikeset et al., 2016; Hutchings & Kuparinen, 2019; Kuparinen et al., 2014). Importantly, reductions in overall population numbers may not necessarily translate into a reduction in local population density if fish continue to group together in schools or aggregations. If there is selection against sociability, however, then localized buffering to changes in overall population density may be less likely to occur. Furthermore, demands on finite resources and corresponding effects on competition and aggression may also interact to reduce any positive effects of localized density maintenance on fishing‐associated selection, but more research is needed to address these issues. It is still crucial to consider such G×E effects to address the complexity of population‐level ecological and evolutionary responses to intense harvesting. As density may thus affect both the evolution and the recovery of the targeted populations, the strategy used to mitigate selection and evolution under one set of circumstances may be ineffective or inappropriate in another.

The reduced density of the population did not change overall behavioural expression compared to the normal density. By changing the social structure of the population (Kavanagh & Olney, 2006), the density reduction was expected to influence activity behaviour and aggression. Previous studies have shown that that, in fish species with social hierarchies, aggression is altered by density (Kavanagh & Olney, 2006), even when the amount of available food is adjusted for group density (Laursen et al., 2013). In addition, fish growth rate can be reduced at higher densities, with greater variance in food intake and growth, even when food availability is increased relative to the increased density (Canario et al., 1998). The absence of difference in overall behaviour in our study (behaviour both from the PCAs and in isolation) revealed that even if changes might have occurred at the individual level depending on the population structure, the overall behaviour of the population remains constant between developmental density conditions.

No indirect selection was observed between body size and any of the behaviours measured in the present study, as no genetic correlation was observed among these traits. It has previously been suggested that fisheries‐induced changes in individual size might be the result of direct selection on behaviour driving evolutionary changes in correlated life‐history traits such as length or growth rate, or vice‐versa, because these traits are assumed to be correlated (Biro & Post, 2008; Sbragaglia et al., 2019; Uusi‐Heikkila et al., 2008). Selection experiments have shown that several generations of size‐based selection can cause an evolutionary change in fish activity, exploration and social behaviour (Diaz Pauli et al., 2019; Diaz Pauli & Sih, 2017; Sbragaglia et al., 2019; Uusi‐Heikkila et al., 2015). Our results do not support the hypothesis of correlated selection between behaviour and size. Even if traits display a phenotypic correlation, this will not necessarily translate into a corresponding genetic correlation, as is the case here between length and distance to conspecifics. Length and behaviour thus have the possibility to be genetically uncorrelated. Therefore, the selection or evolution observed for these traits, instead of being the result of correlated selection, would rather be the result of direct independent selection on both of these traits or other life‐history trade‐offs. As heritability and genetic correlations dependent on context, species or populations (Crespel, Bernatchez, Audet et al., 2013; Crespel, Bernatchez, Garant et al., 2013), such absence of genetic correlation may not be the case for other targeted species, but it would need to be further investigated as so far this aspect has been overlooked. Delineating between correlated and independent selection is crucial to better advise fisheries management.

## 5 CONCLUSIONS

In summary, active fishing gear can induce both direct selection and indirect selection on a wide range of fish behaviours, potentially leading to evolution of the targeted population over time. However, this evolution could be highly context dependent as a reduction in population density would have the potential to shift the evolutionary trajectory. As evolution of fish behaviour could have drastic consequences, it is essential to not only consider operating gear towards selection on a wider range of behaviours, but also to better document the heritability and genetic correlations of the diversity of traits potentially under selection. This would allow us to discriminate among direct selection and indirect selection of the gear, better predict the evolutionary consequences of fishing and to advise more wisely the fishing policies. Considering the fishing selective effect on fish behaviour, size‐based regulations might not be sufficient to mitigate the evolutionary consequences of fishing. In addition, it is necessary to use a more integrative approach combining gear selection but also other harvest‐associated environmental aspects of fishing, such as population density, habitat alteration, prey availability and distribution, within‐ and among‐species competition, and natural predation, to better address the complexity of the ecological and evolutionary consequences of such human‐induced evolutionary pressure on wild populations.

## CONFLICT OF INTEREST

The authors declare no competing interests.

## Data Availability

Data that support the findings of this study are openly available in the public repository Enlighten at (https://doi.org/10.5525/gla.researchdata.1167).
